# Predictive value of the prognostic nutritional index combined with serum chloride levels for the prognosis of patients with acute decompensated heart failure

**DOI:** 10.1007/s00380-024-02381-x

**Published:** 2024-03-19

**Authors:** Wenyi Gu, Yanji Zhou, Baotong Hua, Wenfang Ma, Ling Dong, Tao Shi, Jie Zou, Na Zhu, Lixing Chen

**Affiliations:** Kunming Medical University First Affiliated Hospital, No. 295 Xichang Road, Wuhua District, Kunming, 650032 Yunnan China

**Keywords:** Acute decompensated heart failure, Serum chloride prognostic, Nutritional index, All-cause mortality, Prognosis

## Abstract

**Background:**

The prognostic nutritional index (PNI) and serum chloride level are related to adverse outcomes in patients with heart failure. However, little is known about the relationship between the PNI and serum chloride level in predicting the poor prognosis of patients with acute decompensated heart failure (ADHF).

**Methods and results:**

We reviewed 1221 consecutive patients with ADHF admitted to the First Affiliated Hospital of Kunming Medical University from January 2017 to October 2021. After excluding patients with in hospital death, missing follow-up data, missing chloride data, missing lymphocyte (LYM) count data, or missing serum albumin data, 805 patients were included. PNI was calculated using the formula: serum albumin (ALB) (g/L) + 5 × LYM count (10^9/L). Patients were divided into 4 groups according to the quartiles of the PNI, and the highest PNI quartile (PNI Q4: PNI ≥ 47.3) was set as the reference group. The patients in the lowest PNI quartile (PNI Q1: PNI < 40.8) had the lowest cumulative survival rate, and mortality risk decreased progressively through the quartiles (log-rank *χ*^2^ 142.283, *P* < 0.0001). Patients with ADHF were divided into 8 groups by quartiles of PNI and median levels of serum chloride. After adjustment, the hazard ratio (HR) for all-cause mortality in ADHF patients in Group 1 was 8.7 times higher than that in the reference Group 8. Furthermore, the addition of serum chloride level and PNI quartile to the Cox model increased the area under the Receiver operating characteristic (ROC) curve by 0.05, and the area under the ROC curve of the new model was higher than that of the original model with traditional risk factors.

**Conclusions:**

Both the lowest PNI quartiles and low chloride level indicate a higher risk of all-cause death in patients with ADHF.

## Introduction

Heart failure (HF) is a highly prevalent disease with significant morbidity and mortality worldwide, with an estimated 64.3 million people worldwide suffering from HF in 2017 [1]. The prevalence of HF continues to rise in China. A study showed that the nationally standardized prevalence of HF among people over 25 years of age in China was 1.10%, implying that the total number of people over 25 years of age with HF in China is approximately 12.1 million [2]. The China Heart Failure (China-HF) registry shows that the in-hospital mortality rate due to HF is 4.1% ± 0.3% [3]. The high mortality and readmission rates of HF patients bring about a huge public health burden on the country.

Malnutrition is common in patients with HF [4]. It is estimated that up to 50% of HF patients are malnourished [5]. Due to chronic inflammation, dyspepsia, decreased absorption, excessive decomposition, insulin resistance and other possible mechanisms, the nutritional status of HF patients may deteriorate [6–9]. At the same time, fluid retention and inflammation can be exacerbated by malnutrition, and neuroendocrine factors are activated, accelerating HF and creating a vicious cycle [10]. The mortality, cardiovascular events and repeated hospitalisation rate of undernourished HF patients are higher than those of well-nourished patients [11].Recently, a new simple index, the prognostic nutritional index (PNI), has been used to assess nutritional status in a variety of clinical situations, including cancer and postoperative pneumonia [12, 13]. The PNI is calculated from serum albumin (ALB) concentrations and total lymphocyte (LYM) counts in peripheral blood. Recent studies have also shown that in patients with acute or chronic HF, lower PNI is associated with increased mortality [14, 15].

Serum chloride has many physiological functions in the human body. (1) It is the most important anion outside the cell, accounting for 97–98% of all strong anion charges and two-thirds of all negative charges in plasma; (2) it maintains serum electrical neutrality; (3) it contributes to the production of hydrochloric acid (HCl), the maintenance of osmotic pressure gradient and the secretion of gastrointestinal fluids; (4) it is the main strong ion outside the cell and the key electrolyte for maintaining acid‒base balance; (5) it contributes to general electrical activities (such as muscle and myocardial activities); (6) it affects oxygen transportation and gas exchange; (7) it helps to maintain blood pressure and renal function; and (8) it contributes to the flow of water between body fluids [16–19]. Some studies have shown that low serum chloride levels are an independent predictor of adverse outcomes in patients with acute HF and chronic HF [20–26].

Both malnutrition and low chloride levels are independent prognostic factors in HF patients [14, 27], but the interrelationship between PNI and low serum chloride levels in predicting the risk of all-cause mortality in acute decompensated heart failure (ADHF) patients is unclear. Therefore, this study aims to explore the relationship between the PNI and low serum chloride levels in patients with ADHF.

## Methods

### Study population

We enrolled 1221 patients diagnosed with ADHF admitted to the First Affiliated Hospital of Kunming Medical University from January 2017 to October 2021. We included patients who were admitted with ADHF (NYHA class III or IV) and brain natriuretic peptide (BNP) level of ≥ 500 pg/mL. The study included 805 patients with ADHF, excluding those who died in the hospital, were missing chloride data, did not have LYM count data, were missing serum ALB data or did not have follow-up data.

### Data collection

Demographic and clinical information, electrocardiograms, complications, and medication history were reported on admission. Demographics included age and sex, and clinical data consisted of body mass index (BMI), NYHA cardiac classification, complications, and medical history. Blood samples were taken prior to any therapy measures, including BNP, myoglobin, serum creatine phosphokinase isoenzyme (CK-MB), troponin I and d-dimer. After fasting for 12 h, other blood samples were collected in strict accordance with standard procedures and sent to the laboratory of the First Affiliated Hospital of Kunming Medical University for immediate testing according to standard techniques. Detection indicators included routine blood tests, white blood cell count, LYM count, serum chloride, serum sodium, serum ALB, serum urea, serum creatinine (CRE), serum uric acid (UA), C reactive protein (CRP), haemoglobin (HB), total cholesterol (TC), triglycerides (TG), etc. PNI was calculated using the formula: ALB (g/L) + 5 × LYM count (10 9/L). The estimated glomerular filtration rate (eGFR) was calculated by the Modification of Diet in Renal Disease equation applied to the Chinese cohort. A bedside electrocardiogram (ECG) machine was used to collect the ECG data of patients. Echocardiography was completed within three days of admission. If the patient was readmitted due to worsening HF, we recorded the time of the first readmission and the data. After discharge, the researchers collected survival data from patients with ADHF through telephone interviews. The primary endpoint of this study was all-cause mortality.

### Ethics

The Medical Ethics Committee of the First Affiliated Hospital of Kunming Medical University approved the study, which also complied with the Declaration of Helsinki. Informed consent was obtained from all patients with ADHF included in the study.

### Statistical analysis

Normally distributed continuous variables are expressed as the mean ± standard deviation. Nonnormally distributed variables are expressed as medians and interquartile ranges. Categorical variables are expressed as numbers and percentages. One-way ANOVA and Kruskal–Wallis tests were used for comparisons between group differences in continuous variables. The *χ*^2^ test was used to compare between-group differences in categorical variables. Due to the skewed distribution of BNP levels, it was converted to the natural logarithm. A multivariate Cox proportional hazard model was used to test the risk ratio of independent prediction of all-cause mortality, and covariates selected in advance were included because of their prognostic relevance or the possibility of confusing the risk relationship between chloride and/or PNI. HRs and 95% confidence intervals (CIs) were determined. Survival estimates were analyzed via the Kaplan‒Meier method and log-rank test to investigate the survival curves. ROC analysis was used to estimate the predictive value of serum chloride and PNI for mortality risk in patients with ADHF. The data were statistically analyzed using SPSS 26.0. Bilateral *P*-values < 0.05 were considered statistically significant.

## Results

### Baseline patient characteristics

Baseline characteristics of patients with ADHF in quartile groupings of the PNI are shown in Table 1. A total of 805 patients with ADHF were included and analyzed in this study. The average age was 66.7 ± 12.3 years, including 488 (60.6%) males. Patients were divided into four groups based on interquartile spacing: Q1: PNI < 40.8; Q2: PNI ≥ 40.8 to < 44.2; Q3: PNI ≥ 44.2 to < 47.3, Q4: PNI ≥ 47.3. Patients with higher PNI values may be younger and higher LYM count, higher HB, higher serum ALB, lower CRE, higher eGFR, higher serum sodium, lower CRP, and lower LogBNP compared to patients with lower PNI values.

**Table 1:** Baseline characteristics according to PNI quartile

	Total (*n* = 805)	PNI (quartile)				*P*-value
		Q1 (*n* = 196) (< 40.8)	Q2 (*n* = 203) (40.8–44.1)	Q3 (*n* = 197) (44.2–47.2)	Q4 (*n* = 209) (≥ 47.3)	
Age, years	66.65 ± 12.30	71.35 ± 10.93	66.69 ± 11.98	66.55 ± 11.92	62.30 ± 12.63	*P* < 0.0001
Male sex, *n* (%)	488 (60.6%)	121 (61.7%)	134 (66.0%)	111 (56.3%)	122 (58.4%)	*P* = 0.211
Hypertension, *n* (%)	455 (56.5%)	111 (56.6%)	112 (55.2%)	112 (56.9%)	120 (57.4%)	*P* = 0.973
Diabetes mellitus, *n* (%)	213 (26.5%)	63 (32.1%)	53 (26.1%)	53 (26.9%)	44 (21.1%)	*P* = 0.093
Coronary heart disease, *n* (%)	410 (50.9%)	101 (51.5%)	106 (52.2%)	101 (51.3%)	102 (48.8%)	*P* = 0.908
Smoking, *n* (%)	263 (32.7%)	59 (30.1%)	61 (30.0%)	68 (34.5%)	75 (35.9%)	*P* = 0.475
Drinking, *n* (%)	130 (16.1%)	31 (15.8%)	31 (15.3%)	29 (14.7%)	39 (18.7%)	*P* = 0.706
BMI, kg/m^2^	23.05 ± 3.79	22.32 ± 3.22	23.34 ± 4.06	22.93 ± 3.82	23.57 ± 3.90	*P* = 0.005
Heart rate, bpm	83.90 ± 20.59	83.36 ± 21.23	83.89 ± 19.40	84.63 ± 20.53	83.71 ± 21.27	*P* = 0.940
SBP, mmHg	122.56 ± 21.93	121.76 ± 22.58	124.36 ± 21.11	122.41 ± 20.98	121.72 ± 22.99	*P* = 0.583
DBP, mmHg	77.01 ± 14.79	74.61 ± 14.53	77.47 ± 14.00	76.67 ± 15.31	79.13 ± 15.05	*P* = 0.021
WBC count, 109/L	7.52 ± 3.35	7.69 ± 3.69	7.58 ± 3.57	7.55 ± 3.25	7.28 ± 2.89	*P* = 0.658
LYM count, 109/L	1.47 ± 0.67	1.02 ± 0.44	1.29 ± 0.40	1.57 ± 0.45	1.98 ± 0.85	*P* < 0.0001
HB, g/L	135.5 ± 23.49	124.77 ± 26.09	136.39 ± 22.76	135.90 ± 21.56	144.33 ± 19.14	*P* < 0.0001
ALB, g/L	36.92 ± 4.21	32.42 ± 3.17	35.94 ± 2.08	37.75 ± 2.23	41.31 ± 3.20	*P* < 0.0001
CRE, mmol/L	101.70 (83.00, 131.55)	106.70 (84.88, 147.58)	108.30 (85.50, 136.20)	99.30 (79.65, 124.70)	95.90 (81.40, 118.45)	*P* < 0.0001
UA, umol/L	490.71 ± 162.75	489.49 ± 163.67	494.96 ± 166.34	488.51 ± 158.16	489.75 ± 163.66	*P* = 0.979
GFR, ml/min	44.74 ± 18.23	38.65 ± 17.23	42.99 ± 16.04	46.11 ± 17.04	50.87 ± 20.12	*P* < 0.0001
Chloride, mmol/L	103.30 ± 4.32	102.18 ± 4.70	103.21 ± 4.69	103.88 ± 3.80	103.89 ± 3.82	*P* < 0.0001
Sodium, mmol/L	141.32 ± 4.16	140.47 ± 4.52	141.07 ± 4.17	141.79 ± 4.04	141.91 ± 3.75	*P* = 0.001
CRP, mg/L	7.22 (3.20, 21.93)	14.60 (5.71, 35.19)	7.43 (3.73, 16.80)	6.96 (3.32, 19.79)	4.19 (2.09, 12.18)	*P* < 0.0001
LogBNP	3.15 ± 0.27	3.22 ± 0.29	3.18 ± 0.25	3.12 ± 0.27	3.09 ± 0.26	*P* < 0.0001
LVEF, %	45.55 ± 16.44	47.69 ± 16.94	44.22 ± 15.20	45.95 ± 16.51	44.47 ± 16.95	*P* = 0.128
NYHA functional class IIIeIV, *n* (%)	265 (32.9%)	67 (34.2%)	79 (38.9%)	60 (30.5%)	59 (28.2%)	*P* = 0.108
Pharmacotherapy						
Dapagliflozin, *n* (%)	164 (20.4%)	49 (25.0%%)	37 (18.2%)	44 (22.3%)	34 (16.3%)	*P* = 0.122
ACEI/ARB/ARNI, *n* (%)	434 (53.9%)	107 (54.6%)	119 (58.6%)	112 (56.9%)	96 (45.9%)	*P* = 0.048
Beta-blocker, *n* (%)	615 (76.4%)	144 (73.5%)	154 (75.9%)	159 (80.7%)	158 (75.6%)	*P* = 0.382
Spironolactone, *n* (%)	661 (82.1%)	157 (80.1%)	169 (83.3%)	167 (84.8%)	168 (80.4%)	*P* = 0.555
Furosemide, *n* (%)	720 (89.4%)	174 (88.8%)	188 (92.6%)	175 (88.8%)	183 (87.6%)	*P* = 0.376

Normally distributed continuous variables are expressed as the mean ± standard deviation. Nonnormally distributed variables are expressed as medians and interquartile ranges. Categorical variables are expressed as numbers and percentages. One-way ANOVA and Kruskal–Wallis tests were used for comparisons between group differences in continuous variables. The *χ*^2^ test was used to compare between-group differences in categorical variables. A *P*-value < 0.05 was considered indicative of statistical significance

*BMI* body mass index, *SBP* systolic blood pressure, *DBP* diastolic blood pressure, *WBC* white blood cells, *LYM* lymphocyte, *HB* haemoglobin, *ALB* albumin, *CRE* serum creatinine, *UA* serum uric acid, *GFR* glomerular filtration rate, *CRP* C reactive protein, *BNP* brain natriuretic peptide, *LVEF* left ventricular ejection fraction, *PNI* prognostic nutritional index, *ACEI* angiotensin-converting enzyme inhibitor, *ARB* angiotensin II receptor blocker, *ARNI* angiotensin receptor neprilysin inhibitor

### PNI and risk of all-cause mortality in patients with ADHF

**Fig. 1:**
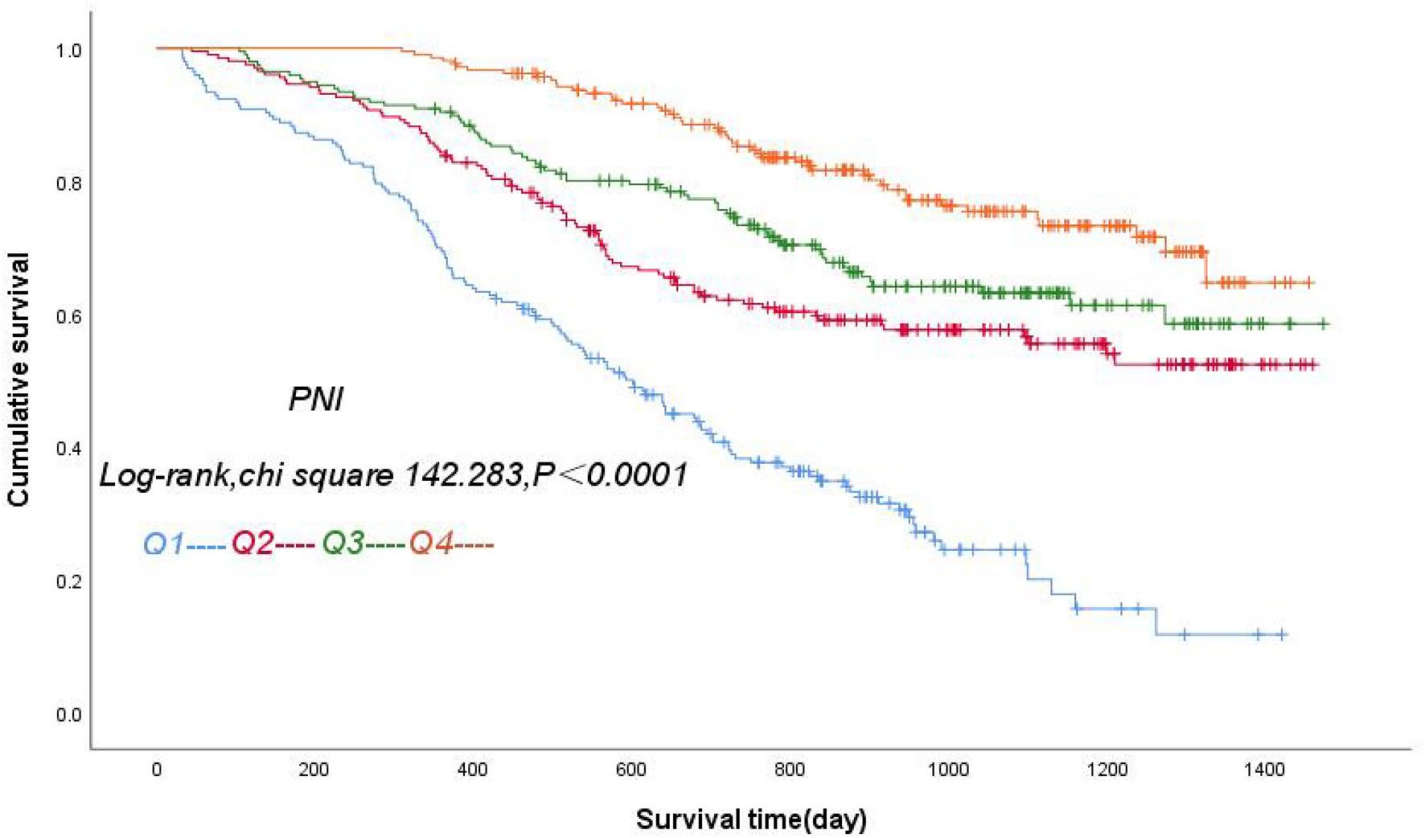
Kaplan‒Meier survival curves for patients with ADHF across PNI quartiles. Note: Q1: PNI<40.8, Q2: PNI≥40.8, PNI<44.2, Q3: PNI≥44.2, PNI<47.3, Q4: PNI≥47.3

**Fig. 2:**
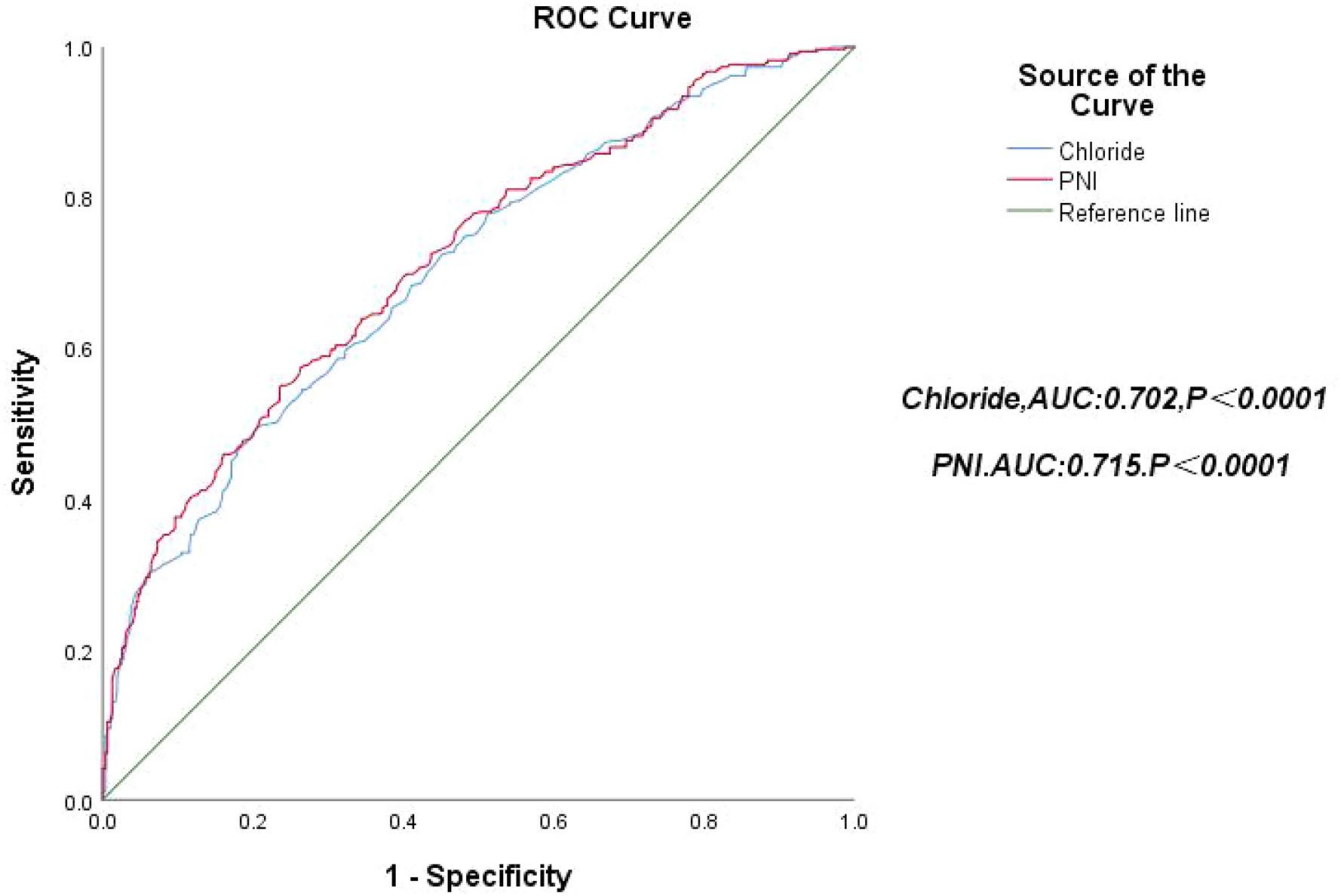
Receiver operating characteristic curve of chloride and PNI for predicting the mortality of patients with ADHF

All 805 patients were divided into 4 groups according to the quartiles of the PNI, and the highest PNI quartile (PNI Q4:PNI ≥ 47.3) was set as the reference group. Figure 1 shows that the patients in the lowest PNI quartile (PNI Q1:PNI < 40.8) had the lowest cumulative survival rate, and mortality risk decreased progressively through the quartiles (log-rank *χ*^2^ 142.283, *P* < 0.0001). The ROC curve for the PNI to predict all-cause mortality in patients with ADHF is shown in Fig. 2 (red line), with an area under the curve of 0.715 (95% CI 0.666–0.738, *P* < 0.0001).

### Serum chloride and risk of all-cause mortality in patients with ADHF

**Fig. 3:**
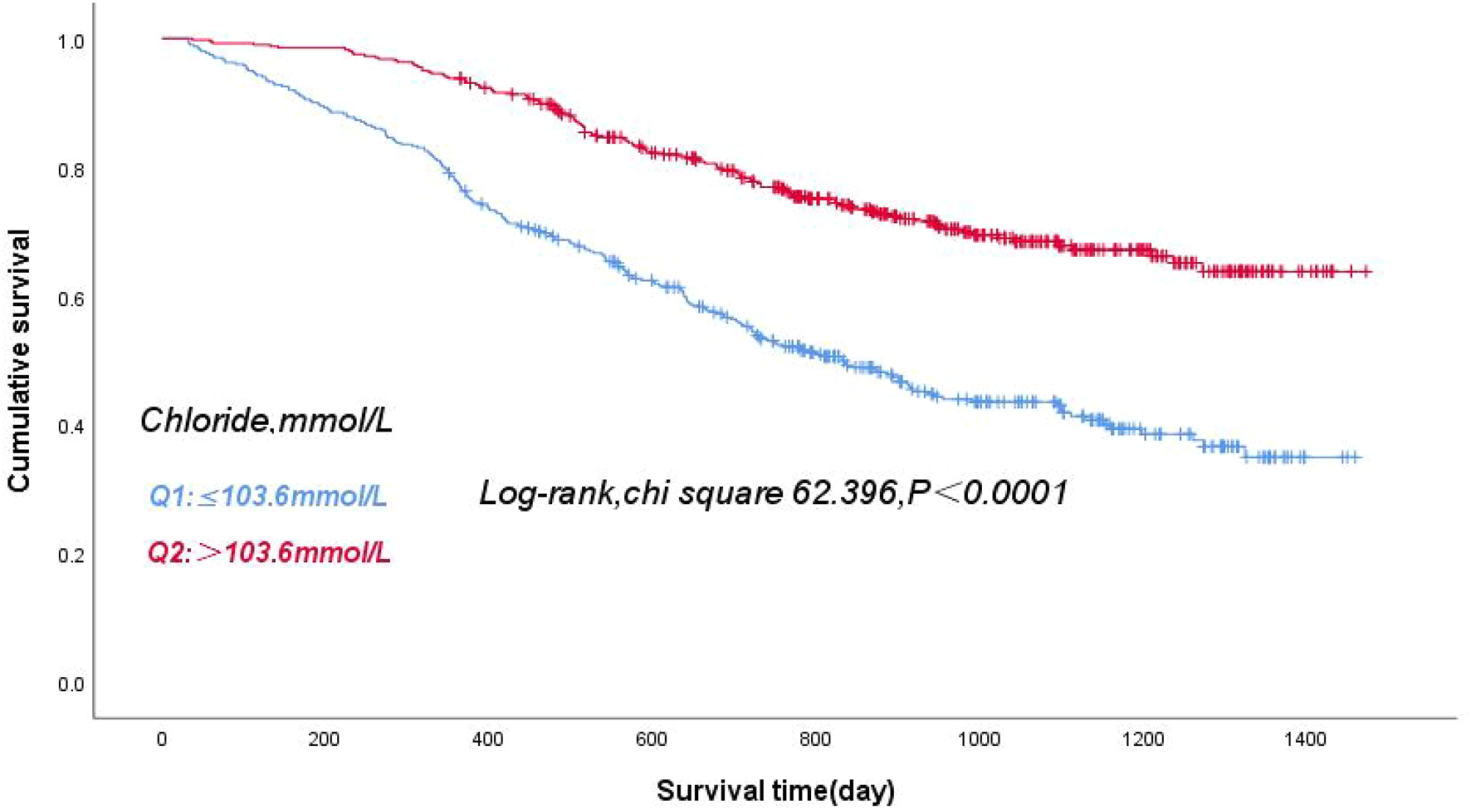
Kaplan‒Meier survival curves for patients with ADHF across median serum chloride levels. Note: Q1: Chloride≤103.6 mmol/L, Q2: Chloride>103.6 mmol/L

Patients were divided into two groups based on the median serum chloride level. Figure 3 shows that the patients in the lowest serum chloride group (Q1:Chloride ≤ 103.6 mmol/L) had the lowest cumulative survival rate (log-rank *χ*^2^ 62.396, *P* < 0.0001). The ROC curve for the use of serum chloride level to predict all-cause mortality in patients with ADHF is shown in Fig. 2 (blue line), with an area under the curve of 0.702 (95% CI 0.680–0.751, *P* < 0.0001).

### PNI combined with serum chloride for predicting the risk of all-cause mortality in patients with ADHF

In the unadjusted Cox analysis, serum chloride level was a predictor of all-cause mortality in patients with ADHF (HR: 0.87, 95% CI 0.85–0.89; *P* < 0.0001) as shown in Table 2. After adjusting for age, sex, coronary heart disease, hypertension, diabetes mellitus, NYHA functional class III e IV, BMI, left ventricular ejection fraction (LVEF), LogBNP, PNI, HB, serum chloride, serum sodium, CRE, UA, GFR and CRP, the serum chloride level was still an independent predictor of all-cause mortality in patients with ADHF (HR: 0.90, 95% CI 0.87–0.93; *P* < 0.0001). As a continuous variable, the risk for all-cause mortality in patients with ADHF decreased by 13% per 1 mmol/L increase in serum chloride level.

**Table 2:** Univariable and multivariable Cox analyses of PNI and serum chloride level predicting all-cause mortality in patients with ADHF

	Univariable		Multivariable	
	HR (95% CI)	*P*	HR (95% CI)	*P*
Age	1.03 (1.02, 1.04)	*P* < 0.0001	1.02 (1.00, 1.03)	*P* = 0.012
Male vs. female	0.98 (0.79, 1.22)	*P* = 0.978		
Coronary heart disease	0.92 (0.74, 1.14)	*P* = 0.428		
Hypertension	0.87 (0.70, 1.08)	*P* = 0.196		
Diabetes mellitus	1.12 (0.88, 1.42)	*P* = 0.358		
NYHA functional class IIIeIV	1.89 (1.53, 2.35)	*P* < 0.0001	1.54 (1.18, 2.01)	*P* = 0.001
BMI	0.92 (0.89, 0.95)	*P* < 0.0001		
LVEF	0.991 (0.984, 0.998)	*P* = 0.009		
LogBNP	5.69 (3.82, 8.50)	*P* < 0.0001	2.97 (1.74, 5.09)	*P* < 0.0001
PNI	0.88 (0.86, 0.89)	*P* < 0.0001	0.91 (0.88, 0.93)	*P* < 0.0001
HB	0.98 (0.97, 0.98)	*P* < 0.0001	0.99 (0.98, 0.99)	*P* < 0.0001
Chloride	0.87 (0.85, 0.89)	*P* < 0.0001	0.90 (0.87, 0.93)	*P* < 0.0001
Sodium	0.91 (0.88, 0.93)	*P* < 0.0001		
Cre	1.002 (1.001, 1.003)	*P* = 0.004		
UA	1.001 (1.000, 1.002)	*P* = 0.002		
GFR	0.972 (0.965, 0.978)	*P* < 0.0001		
CRP	1.009 (1.005, 1.012)	*P* < 0.0001		

Adjusted for age, sex, coronary heart disease, hypertension, diabetes mellitus, NYHA functional class III e IV

*BMI* body mass index, *LVEF* LogBNP, *PNI* prognostic nutritional index, *HB* haemoglobin, chloride, sodium, *CRE* serum creatinine, *UA* serum uric acid, *GFR* glomerular filtration rate, *CRP* C reactive protein

**Fig. 4:**
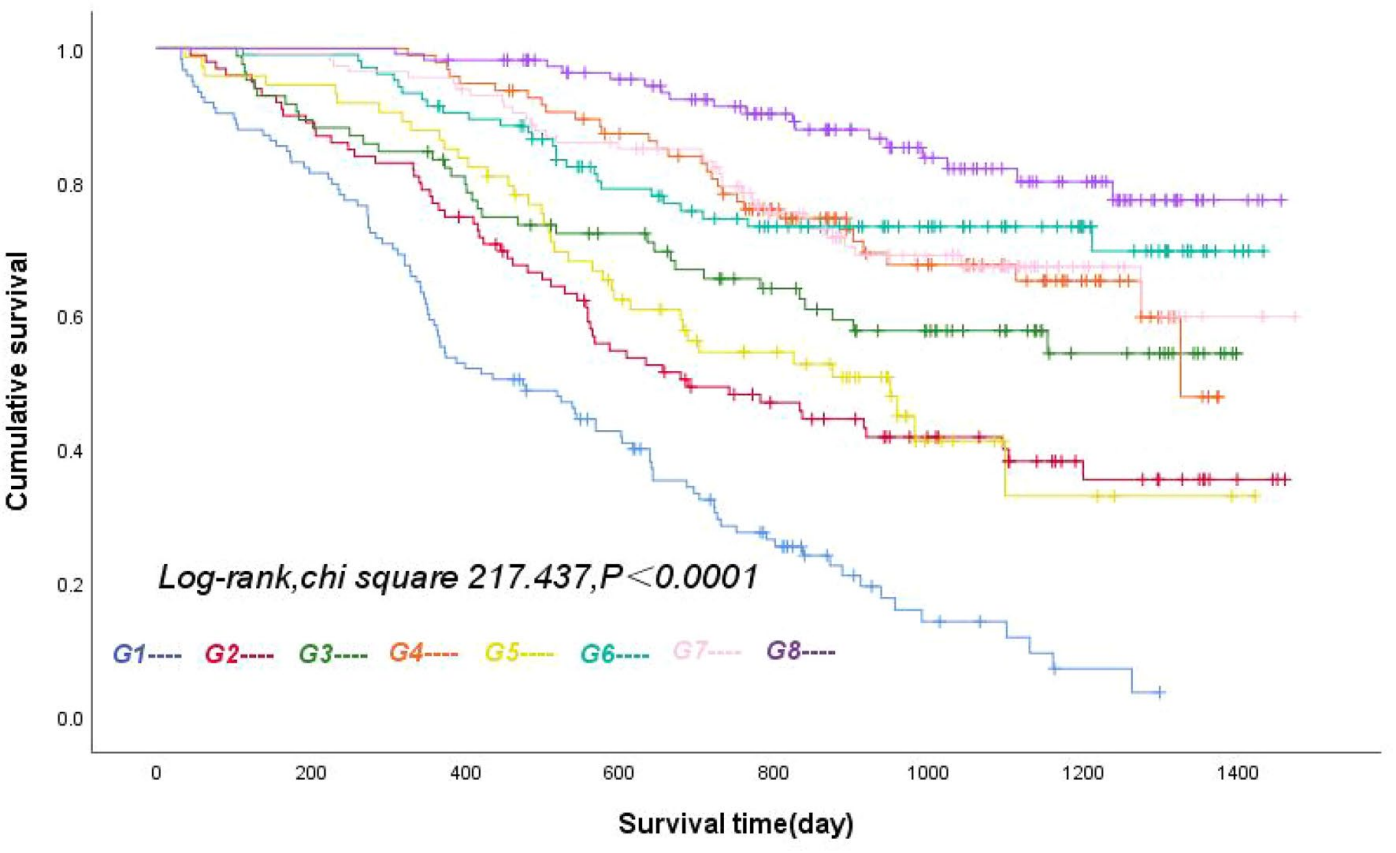
Kaplan‒Meier survival curves for median serum chloride levels and PNI quartiles combined groups. Note: G1:Chloride≤103.6+PNI Q1;G2:Chloride≤103.6+PNI Q2;G3:Chloride≤103.6+PNI Q3;G4:Chloride≤103.6+PNI Q4;G5:Chloride＞103.6+PNI Q1;G6:Chloride＞103.6+PNI Q2;G7:Chloride＞103.6+PNI Q3;G8:Chloride＞103.6+PNI Q4

Patients with ADHF were divided into 8 groups by quartiles of PNI and median levels of serum chloride. As shown in Fig. 4, the Group 1 had the lowest survival rate, while the Group 8 had the highest survival rate. The eighth group (G8:Chloride > 103.6 + PNI Q4) was set as the reference group, and HR values were calculated. As shown in Table 3, after adjusting for age, sex, coronary heart disease, hypertension, diabetes mellitus, NYHA functional class III e IV, BMI, LVEF, LogBNP and CRE, Group 8 was the reference HR for the first group was 8.70 (G1:Chloride ≤ 103.6 + PNI Q1, 95% CI 5.21–14.54, *P* < 0.0001), the HR for the second group was 3.89 (G2:Chloride ≤ 103.6 + PNI Q2, 95% CI 2.28–6.65, *P* < 0.0001), the HR for the third group was 2.67 (G3:Chloride ≤ 103.6 + PNI Q3, 95% CI 1.50–4.76, *P* = 0.001), the HR for the fourth group was 2.20 (G4:Chloride ≤ 103.6 + PNI Q4, 95% CI 1.22–3.96, *P* = 0.009), the HR for the fifth group was 3.72 (G5:Chloride > 103.6 + PNI Q1, 95% CI 2.10–6.58, *P* < 0.0001), the HR for the sixth group was 1.81 (G6:Chloride > 103.6 + PNI Q2, 95% CI 0.99–3.30, *P* = 0.053) and the HR for the seventh group was 1.98 (G7:Chloride > 103.6 + PNI Q3, 95% CI 1.12–3.53, *P* = 0.02). As shown in Table 3, patients in the lowest quartile of PNI and serum chloride Group 1 (Q1:Chloride ≤ 103.6 mmol/L) had an 8.70-fold (95% CI 5.21–14.54, *P* < 0.0001) higher all-cause mortality rate than patients in the highest quartile of PNI and serum chloride Group 2 (Q2:Chloride > 103.6 mmol/L). A lower PNI can increase the HR of ADHF patients with low chloride levels.

**Table 3:** Univariable and multivariable Cox analyses of PNI combined with serum chloride predicting all-cause mortality in patients with ADHF

	Unadjusted		Adjusted	
	HR (95% CI)	*P*	HR (95% CI)	*P*
Chloride (Q2)	Reference		Reference	
Chloride (Q1)	2.40 (1.92, 3.01)	*P* < 0.0001	2.14 (1.71, 2.69)	*P* < 0.0001
PNI (Q4)	Reference			
Q1	5.30 (3.80, 7.38)	*P* < 0.0001	4.17 (2.96, 5.87)	*P* < 0.0001
Q2	2.24 (1.58, 3.20)	*P* < 0.0001	1.89 (1.32, 2.70)	*P* = 0.001
Q3	1.70 (1.17, 2.45)	*P* = 0.005	1.52 (1.05, 2.20)	*P* = 0.029
*Combined categories*				
Group 8:Chloride > 103.6 + PNI Q4	Reference		Reference	
Group 1:Chloride ≤ 103.6 + PNI Q1	10.92 (6.59, 18.11)	*P* < 0.0001	8.70 (5.21, 14.54)	*P* < 0.0001
Group 2:Chloride ≤ 103.6 + PNI Q2	5.36 (3.15, 9.09)	*P* < 0.0001	3.89 (2.28, 6.65)	*P* < 0.0001
Group 3:Chloride ≤ 103.6 + PNI Q3	3.23 (1.82, 5.71)	*P* < 0.0001	2.67 (1.50, 4.76)	*P* = 0.001
Group 4:Chloride ≤ 103.6 + PNI Q4	2.13 (1.19, 3.83)	*P* = 0.011	2.20 (1.22, 3.96)	*P* = 0.009
Group 5:Chloride > 103.6 + PNI Q1	4.68 (2.67, 8.22)	*P* < 0.0001	3.72 (2.10, 6.58)	*P* < 0.0001
Group 6:Chloride > 103.6 + PNI Q2	1.86 (1.03, 3.38)	*P* = 0.041	1.81 (0.99, 3.30)	*P* = 0.053
Group 7:Chloride > 103.6 + PNI Q3	2.07 (1.17, 3.66)	*P* = 0.013	1.98 (1.12, 3.53)	*P* = 0.02

Adjusted for age, sex, coronary heart disease, hypertension, diabetes mellitus, NYHA functional class III and IV

*BMI* body mass index, *LVEF* LogBNP, *CRE* serum creatinine

**Fig. 5:**
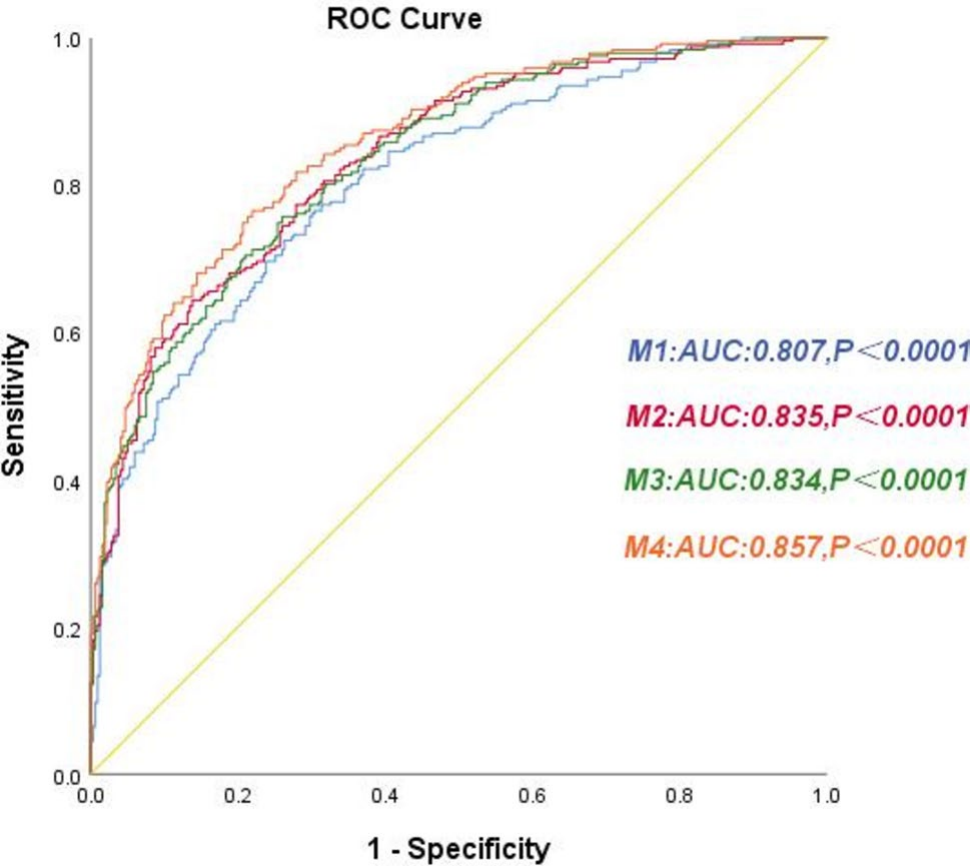
Receiver operating characteristic curve of clinical prediction models in patients with ADHF. Note: Model 1: Adjusted for age, sex, CHD(coronary heart disease),Hypertension, Diabetes mellitus, NYHA functional class III e IV, BMI(body mass index), LVEF, LogBNP, HB(haemoglobin), Sodium, CRE(serum creatinine), UA(serum uric acid), GFR(glomerular filtration rate) and CRP(C reactive protein). Model 2: Model 1 + Chloride. Model 3: Model 1 + PNI. Model 4: Model 1 + Chloride + PNI

As shown in Fig. 5, the area under the ROC curve for the original Model 1 using traditional risk factors was 0.807. In contrast, the area under the ROC curve for Model 2 increased to 0.835 with the addition of the median serum chloride level. The area under the ROC curve increased to 0.834 in Model 3 with the addition of PNI quartiles. Compared to Model 1, the area under the ROC curve for Model 4 increased to 0.857 with the addition of median serum chloride levels and PNI quartiles.

## Discussion

In this study, we investigated the relationship of serum chloride levels and PNI in predicting all-cause mortality in patients with ADHF. To our knowledge, we demonstrated for the first time that combining serum chloride levels and PNI categories improved the predictive value of all-cause mortality. The HR for all-cause mortality in ADHF patients in Group 1 was 8.7 times higher than that in the reference Group 8. Furthermore, the addition of serum chloride level and PNI quartile to the Cox model increased the area under the ROC curve by 0.05, and the area under the ROC curve of the new model was higher than that of the original model with traditional risk factors. These findings suggest that PNI combined with serum chloride could provide important prognostic information for the risk stratification of patients with ADHF.

Recent studies have found that PNI, a new and simple, novel prognostic indicator for the assessment of conditions including cancer, is associated with an increased risk of all-cause mortality or major adverse cardiac events (MACEs) in patients with acute or chronic HF [13–15, 28]. The present study found that the PNI was independently associated with all-cause mortality in patients with ADHF. Due to gastrointestinal oedema, anorexia, abnormal liver function and high catabolism induced by cytokines, patients with ADHF often show reduced nutrient intake or insufficient absorption [29, 30]. Gastrointestinal oedema reduces the intake and absorption of proteins, lipids, and vitamins. Liver congestion reduces ALB synthesis, lipid transport, and synthesis, which can cause metabolic disorders [31]. Because malnutrition can cause many serious complications in patients with ADHF, it can directly affect their quality of life and disease prognosis. Therefore, the evaluation of nutritional status is essential in patients with ADHF. Several studies used BMI, controlling nutritional status (CONUT) score, geriatric nutritional risk index (GNRI), and other indicators to assess nutritional status [32]. Although it is convenient to measure BMI in the clinic, BMI is not an ideal index of the nutritional status of HF patients because patients with ADHF usually have water and sodium retention, which leads to short-term changes in body mass, so BMI cannot distinguish the weight caused by excessive fluid and fat. In HF patients, compared with patients with a healthy weight, patients with a higher BMI have a lower risk of all-cause mortality, a phenomenon called the "obesity paradox" [33, 34]. The CONUT score is calculated from serum ALB, LYM count, and cholesterol, which may be confounded by the broad use of statins in patients with HF. The GNRI requires body weight for calculation, which will be confounded by fluid overload status and dramatic changes in HF treatment during hospitalisation. PNI is a simple index that can be easily calculated using the serum ALB level and LYM count, and these indices can be obtained in most clinical laboratories. The decrease in serum ALB levels may be influenced by malnutrition or liver and kidney dysfunction [29]. Recent studies have shown that hypoproteinemia has been used as an indicator of inflammation, cachexia and malnutrition in patients with ADHF [35, 36]. Nutrition, inflammation, liver and kidney diseases and volume status of patients with HF can affect serum ALB levels. Ineffective diuresis and fluid overload may dilute serum ALB, and then lead to worse outcome [37]. Another cause of hypoproteinemia may be liver dysfunction caused by venous congestion and ischemic injury [38]. Other mechanisms include renal insufficiency [39], protein-loss enteropathy secondary to gastrointestinal congestion [40], and hemodilution [41]. LYMs are a standard biomarker of nonspecific inflammation, which is significantly reduced in many advanced diseases, such as HF [42]. Therefore, PNI is a simple and effective tool to assess the nutritional status of patients with ADHF, not only as an indicator of malnutrition but also as an indicator of pro-inflammatory status, which reflects the severity of the disease and may serve as an independent risk factor for the prognosis of all-cause mortality.

We found that low serum chloride levels were independently associated with all-cause mortality in patients with ADHF, consistent with the study by Grodin et al. [21]. Low serum chloride levels are a common electrolyte abnormality in patients with ADHF. The main reason for low serum chloride levels is related to the loss of chloride anions in the gastrointestinal tract or kidneys. Dietary intake and intestinal absorption and excretion are important aspects that need to be considered in the gastrointestinal tract. Intestinal absorption in patients with ADHF can be impaired through congestion of the splanchnic circulation and subsequent intestinal wall oedema and barrier dysfunction [43]. Electrolytes are very important for heart signal transduction and contribute to the cell excitability of the cardiovascular system. The activation of cardiac chloride channels affects the duration of the membrane potential and action potential of the sinoatrial node, which can lead to arrhythmia [34]. Adaptive remodelling of chloride channels can promote myocardial hypertrophy and subsequent progression of HF [44]. More importantly, chloride plays an important role in fluid homeostasis, neurohormone activation and diuretic resistance [45, 46], which are generally considered the main factors for the occurrence and development of HF.

In our study, all-cause mortality was found to be 2.14 times higher in ADHF patients in the low serum chloride level group (Chloride Q1) than in the high serum chloride level group (Chloride Q2). The risk of all-cause mortality in ADHF patients in the low serum chloride level plus low PNI group (G1:Chloride ≤ 103.6 + PNI Q1) was 8.7 times greater than that in the high serum chloride level plus high PNI group (G8:Chloride > 103.6 + PNI Q4). Our finding that the combination of low serum chloride level and PNI could be more prognostic may suggest that nutritional status should also be considered in clinical research focusing on treatment options for patients with low serum chloride level. Electrolyte disorder and malnutrition should also be important treatment targets for patients with ADHF. Effective intervention measures to improve serum chloride levels and nutritional status, such as using drugs to restore digestion and absorption function when the gastrointestinal tract is congested, may provide opportunities for improving the prognosis of ADHF patients.

## Conclusion

Both the lowest PNI quartiles and low chloride indicate a higher risk of all-cause death in patients with ADHF. The combination of serum chloride level and PNI category enhanced the predictive value for all-cause mortality in patients hospitalized for ADHF.

## Limitations

Nevertheless, this study also has some limitations. First, this was a single-centre, retrospective and observational study, and as such, there may have been unmeasured variables that could have affected the results. Second, we mainly investigated patients with an NYHA class of III or IV for enrolment; thus, these results may not be applicable to populations with mild or moderate HF symptoms. Third, we only collected PNI and serum chloride concentrations at admission and, therefore, could not examine the relationship between dynamic changes in these two variables and prognosis. Fourth, the PNI cannot distinguish patients with overnutrition. The relationship between overnutrition and the prognosis of ADHF needs further study.

## Data availability statement

All data generated or analyzed during this study are included in this article. Further enquiries can be directed to the corresponding author.
